# The Determinants of COVID-19-Related Stress Among Caregivers of Individuals at High Risk During the Pandemic

**DOI:** 10.7759/cureus.54538

**Published:** 2024-02-20

**Authors:** Cansu Ünsal, Esra Yalım, Ibrahim Gündoğmuş

**Affiliations:** 1 Psychiatry, Silifke State Hospital, Mersin, TUR; 2 Psychiatry, Çankırı State Hospital, Çankırı, TUR; 3 Psychiatry, Ankara Etlik City Hospital, Ankara, TUR

**Keywords:** stress, risk, pandemic, covid-19, caregiver

## Abstract

Background: Informal caregivers offer a range of support-physical, emotional, and social-to individuals under their care, thereby exposing themselves to potential mental health risks. During the outbreak of COVID-19, caregivers have emerged as a demographic particularly vulnerable to mental health issues owing to their caregiving roles. The aim of the study is to identify the determinants influencing COVID-19-related stress among caregivers of individuals at elevated risk of coronavirus infection.

Materials and Methods: A cross-sectional study was undertaken, utilizing a sample of 1,556 participants who were enlisted via social media and an online survey questionnaire. Participants provided sociodemographic data and completed both the Depression Anxiety Stress Scale (DASS-21) and the COVID-19 Stress Scale (CSS) to assess their mental health status.

Results: The mean age of the participants was 30.76±6.97 years. Of these, 42.35% (n = 659) resided with individuals at high risk for COVID-19, and 72.75% were female. Statistically significant differences were observed in DASS-21 subscale scores as well as in CSS scores for contamination, socioeconomic consequences, traumatic stress, perceived danger, compulsive checking, xenophobia, and total scores between those living and not living with COVID-19 high-risk individuals. Factors such as residing with a COVID-19 high-risk individual, education level, and DASS-21 subscale scores were identified as significant predictors of CSS scores.

Conclusion: The study reveals those caregivers for individuals at high risk for COVID-19 experience elevated levels of depression, anxiety, stress, and COVID-19-related stress. Factors such as living with a high-risk individual, educational level, and mental health status were significant predictors of COVID-19-related stress. Further research is needed to evaluate the mental well-being of caregivers and to develop effective interventions.

## Introduction

Informal caregivers are individuals who assume responsibility for meeting their daily needs and providing medical care to family members with chronic illnesses or cognitive impairments [1]. Prior research has shown that such caregivers often experience adverse mental health effects, including depression, anxiety, and burnout [2-4]. According to the established stress-health process model, stressors related to caregiving and insufficient coping mechanisms can adversely affect psychological well-being by disrupting psychological, emotional, and behavioral factors [5]. These caregiving-associated stressors may also give rise to familial and occupational difficulties, strain interpersonal relationships, and result in negative financial consequences [6]. Consequently, this creates a complex issue affecting both the caregivers and the well-being of the individuals under their care [1].

The COVID-19 outbreak has had deleterious effects on both physical and psychological well-being [7,8]. During this period, widespread fears of infection, concerns about contaminated surfaces, and proximity to potential carriers have been common. Moreover, some individuals adopted compulsive behaviors such as incessant safety checks, while others exhibited symptoms of traumatic stress [9]. This collective psychological reaction has been termed COVID-19 stress syndrome and is associated with emotional distress, avoidance behaviors, poor coping strategies, depression, and anxiety [7,9,10].

During the pandemic, protective measures such as social isolation and quarantine have disproportionately affected caregivers of medically vulnerable individuals [11]. These caregivers have experienced elevated levels of stress and burnout, largely due to the heightened risk of severe illness for older adults and those with comorbidities, the scarcity of vaccinations, and difficulties in accessing healthcare services [6]. Furthermore, the apprehension of transmitting the virus to vulnerable individuals, the stress of potentially falling ill themselves, and the possibility of separation due to severe illness or death have exacerbated mental health problems, including post-traumatic stress disorder, anxiety, depression, and emotional exhaustion among informal caregivers [12,13].

Existing literature inadequately addresses the mental health challenges confronting informal caregivers living with individuals at elevated risk for COVID-19 infection, including conditions such as COVID-19 stress syndrome [6,12]. Therefore, it is crucial to evaluate the psychological well-being of caregivers and implement suitable interventions to not only safeguard their mental health but also to optimize the care provided to recipients. The purpose of this study is to assess and compare the levels of depression, anxiety, and stress among informal caregivers and non-caregivers, as well as to investigate their predictive influence on COVID-related stress levels.

## Materials and methods

Participants and procedure

The study employed a cross-sectional design, encompassing a sample of 1,556 individuals who successfully completed an online questionnaire. Data collection occurred between December 1, 2020, and December 31, 2020, through social media platforms. Eligible participants were aged 18 to 65 and demonstrated adequate cognitive functioning. Furthermore, participants had no documented history of psychiatric illness, were well-informed about COVID-19 within the past two weeks, and had volunteered to partake in the research. Out of 1,832 initial volunteers, 216 were disqualified due to psychiatric history, and 60 failed to complete the study.

Upon invitation, interested participants completed an online consent form voluntarily. The study identified specific groups as particularly vulnerable to COVID-19 infection, including children, the elderly, and individuals with a range of pre-existing physical illnesses such as diabetes, obesity, and chronic diseases [14]. Based on these criteria, the participant pool was divided into two cohorts: individuals cohabiting with persons at high risk for COVID-19 and those who were not.

Data collection

Sociodemographic Data Form: To align with the study’s objectives, a questionnaire was developed by the researchers. This questionnaire gathers information on various sociodemographic factors.

The Depression Anxiety Stress Scale-21 (DASS-21): This psychometric tool was created to measure levels of depression, anxiety, and stress in respondents [15]. The scale is composed of 21 items distributed evenly across three subscales, each containing seven items. The survey responses are recorded using a four-point Likert scale, which ranges from 0 (never) to 3 (always). The tool has Cronbach’s alpha values of α = 0.87, α = 0.85, and α = 0.81 for the depression, anxiety, and stress subscales, respectively in a Turkish clinical sample [16]. In this study, the Cronbach alpha values are 0.91 for depression, 0.89 for anxiety, and 0.86 for stress.

COVID Stress Scale (CSS): The primary objective of this self-report instrument is to evaluate stress levels that are uniquely related to the COVID-19 outbreak [9]. The scale includes 36 items, organized into six unique subscales: danger, socioeconomic consequences, xenophobia, contamination, traumatic stress, and compulsive checking [9]. The scoring system for items is five points. The total scores can vary between 0 and 144, while each subscale has a potential range of 0 to 24. Higher scores indicate greater stress levels. The scale’s validity and reliability were confirmed in a Turkish sample. Cronbach alpha coefficients of the subscales were found as 0.849 for danger, 0.896 for socioeconomic consequences, 0.916 for xenophobia, 0.920 for contamination, 0.882 for traumatic stress, and 0.804 for compulsive checking [7]. In this study, the Cronbach alpha value for CSS is 0.95.

Data analyses

The statistical analyses were conducted using SPSS version 22.0, which is a software developed by IBM Inc. of Chicago, IL, USA. The Kolmogorov-Smirnov test was utilized to verify the normality of the data distribution, and the values of skewness and kurtosis were also examined. For continuous data, descriptive statistics were applied to provide the mean and standard deviation, while frequency and percentage were utilized to represent categorical variables. To compare categorical data between the two groups, Pearson’s Chi-square test was employed. For continuous variables, Student’s t-test was applied after verifying that the data met the parametric assumptions. The determinants of CSS were found using a linear regression model. A statistically significant p-value was defined as one that was less than 0.05.

## Results

In the sample, 42.35% (n = 659) of participants lived with an individual at high risk for COVID-19, whereas the remaining 57.65% (n = 897) did not. A significant proportion of the individuals involved in the study were of the female gender (72.75%), with an average age of 30.76±6.97 years. Table 1 provides a comprehensive comparison of sociodemographic characteristics between those living with and without high-risk individuals for COVID-19. Notably, there were notable differences between the two groups in terms of living circumstances (χ² = 279.818, p < 0.001), gender (χ² = 11.613, p = 0.001), financial status (χ² = 20.019, p < 0.001), and alcohol usage (χ² = 8.770, p = 0.003). Age, marital status, and smoking behaviors did not differ in a way that was statistically significant.

**Table 1:** Comparison of sociodemographic variables of participants between study groups. ꭓ^2^=Pearson Chi-square test, t= student t-test

Variable	Living with someone at risk of COVID-19		Statistic	df	p
	Yes (n=659)	No (n=897)			
Age, year, mean±SD	30.88±7.16	30.67±6.83	t=-0.561	1545	0.575
Gender, n (%)			ꭓ^2^=11.613	1	0.001
Female	509 (77.2)	623 (69.5)			
Male	150 (22.8)	274 (30.5)			
Education, n (%)			ꭓ^2^=6.710	2	0.035
High school	83 (12.6)	79 (8.8)			
University	330 (50.1)	448 (49.9)			
Postgraduate	246 (37.3)	370 (41.2)			
Marital Status, n (%)			ꭓ^2^=5.712	2	0.057
Single	329 (49.9)	393 (43.8)			
Married	296 (44.9)	453 (50.5)			
Other	34 (5.2)	51 (5.7)			
Income status, n (%)			ꭓ^2^=20.019	2	<0.001
Low	135 (20.5)	128 (14.3)			
Middle	255 (38.7)	307 (34.2)			
High	269 (40.8)	462 (51.5)			
Life with, n (%)			ꭓ^2^=279.818	2	<0.001
Alone	0	304 (33.9)			
Family	611 (92.7)	536 (59.8)			
Friends/Other	48 (7.3)	57 (6.4)			
Smoking, n (%)			ꭓ^2^=2.077	1	0.150
Yes	506 (76.8)	660 (73.6)			
No	153 (23.2)	237 (26.4)			
Alcohol, n (%)			ꭓ^2^=8.770	1	0.003
Yes	428 (64.9)	516 (57.5)			
No	231 (35.1)	381 (42.5)			

Psychometric assessments between the two groups are detailed in Table 2. Participants living with individuals at high risk for COVID-19 scored significantly higher on the DASS-21 total and subscales compared to those living without high-risk individuals for COVID-19. Similar consequences were also observed for the CSS total and subscale scores.

**Table 2:** Comparison of psychometric measurement of participants between study groups. CSS: COVID stress scales; DASS-21: Depression Anxiety Stress Scales 21

Variable	Living with someone at risk of COVID-19		t value	df	p
	Yes (n=659)	No (n=897)			
COVID Stress Scales, mean±SD					
Danger	14.77±5.15	13.33±4.75	-5.699	1554	<0.001
Socioeconomic Consequences	5.15±5.43	4.51±4.78	-2.406	1307	0.016
Xenophobia	10.87±6.78	8.48±6.65	-6.930	1554	<0.001
Contamination	13.78±5.77	11.36±6.14	-7.925	1463	<0.001
Traumatic Stress	7.45±5.39	4.85±4.74	-9.865	1308	<0.001
Compulsive Checking	10.56±5.35	9.00±4.80	-5.906	1326	<0.001
CSS Total	62.59±25.30	51.56±24.08	-8.738	1554	<0.001
DASS-21, mean±SD					
Anxiety	5.30±3.53	3.85±3.20	-8.275	1329	<0.001
Depression	7.06±4.33	6.25±4.34	-3.622	1550	<0.001
Stress	7.71±3.89	6.52±3.58	-6.144	1337	<0.001

Subsequent multiple linear regression analysis aimed to ascertain the predictive power of variables such as age, gender, living with a COVID-19 high-risk individual, education level, marital status, income status, and DASS-21 anxiety, depression, and stress scores on the overall CSS score. The analysis yielded a significant relationship among some of these variables and the CSS score (R = 0.531, R² = 0.282, F (12,1517) = 49.754, p < 0.001). According to the model, living with a COVID-19 high-risk individual (p < 0.001), education level (p < 0.001), DASS-21 anxiety (p < 0.001), depression (p < 0.001), and stress (p = 0.049) scores were significant predictors for the CSS score. The details of the linear regression model are presented in Table 3.

**Table 3:** Linear regression results of COVID Stress Scale predictors.

Predictor	B	SE	β	t	p
Intercept	46.998	3.616		12.998	
Age (year)	-0.077	0.082	-0.021	-0.932	0.352
Gender (male)	-1.514	1.245	-0.027	-1.216	0.224
Living with someone at risk of COVID-19 (yes)	5.679	1.145	0.112	4.962	<0.001
Education (High school)					
University	-4.593	1.904	-0.118	-2.413	0.016
Postgraduate	-11.724	1.980	-0.278	-5.920	<0.001
Marital Status (Single)					
Married	-0.165	1.166	-0.005	-0.141	0.888
Other	7.039	2.490	1.057	2.827	1.000
Income status (Low)					
Middle	-2.903	1.646	-0.398	-1.763	0.078
High	-0.738	1.623	-0.128	-0.455	0.649
Depression Anxiety Stress Scales-21					
Stress	1.595	0.265	0.443	6.017	<0.001
Anxiety	2.108	0.246	0.041	8.582	<0.001
Depression	-0.389	0.197	-0.007	-1.968	0.049

## Discussion

The aim of this study was to assess the mental health issues among caregivers cohabiting with individuals at elevated risk for infection-related morbidity and mortality amid the COVID-19 pandemic. Furthermore, the study explored the predictive value of these mental health variables on COVID-19-related stress. Our findings indicate that caregivers of this at-risk cohort exhibit elevated levels of depression, anxiety, and stress. Factors such as cohabitation with high-risk individuals, educational level, and the aforementioned mental health issues were identified as predictors of COVID-19-related stress levels.

Caregiver stress is predominantly typified by psychological indicators, encompassing feelings of being overwhelmed or abandoned, and a proclivity toward social isolation. Additionally, it correlates with physical morbidity, disturbance of family and occupational routines, as well as financial challenges [6]. Previous study has connected providing care to a range of mental health issues, such as anxiety and sadness [17]. During the pandemic of COVID-19, caregivers of children with primary immunodeficiency-who are inherently at higher risk of infection-reported heightened levels of anxiety and post-traumatic stress [13]. Similarly, caregivers of children with chronic kidney disease were found to experience elevated levels of anxiety, depression, stress, and insomnia [18]. In the study conducted by Cohen et al., involving 835 informal caregivers, a relationship was found between caregiving intensity and burnout. It was also noted that this association operates within a complex framework influenced by gender-related factors [19]. A nationwide multicenter study led by Zucca et al., involving 4710 caregivers of dementia patients, revealed elevated levels of stress within this cohort. Notably, anxiety, feelings of being overwhelmed, and a sense of helplessness were prevalent among approximately half and one-third of caregivers, respectively. Additionally, it was reported that depression, anguish, irritability, and sentiments of isolation and abandonment affected between 20 and 30% of respondents [6]. Similarly, in a study conducted by Ismail et al. involving 272 caregivers of patients diagnosed with type 1 diabetes mellitus, it was determined that these individuals experienced higher levels of overall stress [20]. Furthermore, healthcare professionals have not been immune to chronic stress during this period [21,22]. Previous studies revealed that healthcare providers who were involved in direct patient care faced significant levels of fear and anxiety, compounded by concerns of potentially transmitting the virus to their family members, such as children and the elderly [23].

Our study corroborates that caregivers residing with individuals at risk for severe COVID-19 outcomes experience elevated levels of anxiety, depression, and stress. These caregivers also scored higher on both total and subscale measures of COVID-19-related stress. Several factors may account for this, including the detrimental effects of isolation and quarantine protocols, diminished social support compared to pre-pandemic levels, the economic repercussions of the pandemic, and increased caregiving responsibilities [24]. This is further exacerbated by the stress associated with the potential risk of infecting and harming the individuals under their care [23]. Living with individuals who require care is linked to heightened perceived stress, primarily due to fears of illness and the potential disruption of daily activities [1]. In line with this, the present study has shown that residing with high-risk individuals serves as a predictor of COVID-19-related stress levels. Such stress is likely influenced by caregivers’ worries about contracting the virus themselves and the difficulties they face in providing adequate care for their dependents.

Our findings also demonstrate that caregivers with higher educational levels tend to have improved economic stability, which enables them to better manage the needs of those they care for and cope with stress-inducing events [25]. A significant relationship was established between lower educational attainment, informal caregiving roles, and key elements contributing to COVID-19-related stress, such as fear and traumatic stress. The current study corroborates that individuals with lower levels of education are more prone to experiencing COVID-19-related stress.

Moreover, pre-existing anxiety disorders were found to elevate COVID-19-related stress levels [26]. Prior research involving older adults has shown that anxiety levels before the pandemic were predictive of stress induced by COVID-19 [27]. Additionally, our research found a link between severe stress brought on by COVID-19 and other mental health problems, such as anxiety, sadness, fear of infection or death, and money worries [28]. These findings highlight the need for targeted mental health evaluations and interventions for individuals who are especially vulnerable due to the combined stressors of a pandemic and daily obligations.

In line with societal gender roles, women are more likely than men to serve as caregivers, often dedicating more time to the care recipient, taking on more responsibilities, and experiencing higher levels of burnout [25]. Consistent with this, our study revealed that a higher proportion of women than men live with individuals at high risk for COVID-19 infection and assume caregiving roles. Furthermore, our data show that caregivers cohabiting with individuals at risk for COVID-19 infection have significantly higher alcohol consumption compared to non-caregivers. This observation aligns with earlier research suggesting a potential relationship between elevated depression and anxiety levels and increased alcohol use. The results support the idea that caregivers facing social isolation, burnout, and multifaceted stressors, may resort to alcohol as a coping strategy [29].

## Conclusions

In summary, our findings underscore the critical importance of conducting systematic assessments of the mental health status of individuals residing with those at heightened risk of infection, especially those who undertake caregiving roles. The implications extend beyond mere recognition to actionable steps aimed at bolstering their well-being. Enhanced social support mechanisms should be proactively facilitated, acknowledging the unique stressors and challenges faced by caregivers in these circumstances. Moreover, the implementation of targeted psychological interventions is imperative to address the multifaceted needs of this population effectively.

Nevertheless, our study represents a preliminary step in understanding the mental health dynamics of caregivers in the context of heightened infection risk. Further research endeavors are warranted to delve deeper into the complexities of caregivers' experiences and to identify tailored interventions that resonate with their specific needs and circumstances. These future studies should adopt longitudinal designs to capture the dynamic nature of caregiving-related stressors and mental health outcomes over time. Additionally, exploring the differential impact of various support strategies and interventions will contribute to the development of evidence-based approaches that can meaningfully alleviate caregiver distress and enhance their resilience.

In conclusion, while our study sheds light on the mental health challenges faced by caregivers in high-risk environments, it also underscores the ongoing imperative to expand our understanding and enhance support systems for this vulnerable population. By prioritizing comprehensive assessments and targeted interventions, we can cultivate a more supportive and resilient caregiving ecosystem amidst the uncertainties of the pandemic and beyond.
